# Mesencephalic origin of the rostral Substantia nigra pars reticulata

**DOI:** 10.1007/s00429-014-0980-9

**Published:** 2015-01-13

**Authors:** M. Pilar Madrigal, Juan A. Moreno-Bravo, Jesús E. Martínez-López, Salvador Martínez, Eduardo Puelles

**Affiliations:** 1 Instituto de Neurociencias de Alicante, Universidad Miguel Hernández-CSIC, 03550 Sant Joan d’Alacant, Alicante Spain; 2 Instituto Murciano de Investigación Biomédica IMIB-Arrixaca, E30120 Murcia, Spain

**Keywords:** Substantia nigra pars reticulata, Tangential migration, GABAergic neurons, *Nkx6.2*, *Six3*

## Abstract

In embryonic development, the neurons that will constitute a heterogeneous nucleus may have distinct origins. The different components of these populations reach their final location by radial and tangential migrations. The Substantia nigra pars reticulata (SNR) presents a high level of neuronal heterogeneity. It is composed by GABAergic neurons located in the mes-diencephalic basal plate. These inhibitory neurons usually display tangential migrations and it has been already described that the caudal SNR is colonized tangentially from rhombomere 1. Our aim is to unveil the origin of the rostral SNR. We have localized a *Nkx6.2* positive ventricular domain located in the alar midbrain. *Nkx6.2* derivatives’ fate map analysis showed mainly a rostral colonization of this GABAergic neuronal population. We confirmed the mesencephalic origin by the expression of *Six3*. Both transcription factors are sequentially expressed along the differentiation of these neurons. We demonstrated the origin of the rostral SNR; our data allowed us to postulate that this nucleus is composed by two neuronal populations distributed in opposite gradients with different origins, one from rhombomere 1, caudal to rostral, and the other from the midbrain, rostral to caudal. We can conclude that the SNR has multiple origins and follows complex mechanisms of specification and migration. Our results support vital information for the study of genetic modifications in these extremely complex processes that result in devastating behavioral alterations and predisposition to psychiatric diseases. Understanding the development, molecular identity and functional characteristics of these diverse neuronal populations might lead to better diagnosis and treatment of several forms of neurological and psychiatric disease.

**Electronic supplementary material:**

The online version of this article (doi:10.1007/s00429-014-0980-9) contains supplementary material, which is available to authorized users.

## Introduction

The Substantia nigra (SN) is a complex nucleus located not only in the mesencephalic basomedial territory (Puelles 2007; Moreno-Bravo et al. 2012; Puelles et al. 2012) but it is also extended along the pretectum, thalamus and prethalamus (diencephalic prosomeres). It is divided into a pial superficial part, SN pars reticulata (SNR), constituted by GABAergic neurons (GABAn) and a more internal SN pars compacta (SNC), primarily containing dopaminergic neurons (Hanaway et al. 1970). There is, nonetheless, some intermixing of dopamine neurons within the SNR (González-Hernández and Rodríguez 2000). The dopaminergic neurons are a deeply studied population due to their implication in several motor syndromes such as Parkinson’s disease; however, the molecular diversity and regulation of GABAn development are only beginning to be understood. These GABAn control several aspects of behavior, play important roles in psychiatric diseases, susceptibility to drugs of abuse and are also important targets for several medical treatments for these diseases (Jhou et al. 2009; Vargas-Perez et al. 2009; Cohen et al. 2012).

The SNR and the internal segment of the globus pallidus provide the major output projections of the basal ganglia system where the final stage of information processing takes place. These cell groups are mainly composed of GABAn; they integrate inputs from all other components of the basal ganglia system (striatum, globus pallidus, subthalamic nucleus) and elaborate the message sent by this system to extrinsic structures (Rinvik et al. 1976). For this purpose, SNR neurons project to the superior colliculus, reticular formation and thalamus, mainly to the ventral lateral and ventral anterior region. SNR GABAn also issue local axon collaterals that carry out an important role of inhibition within the SNC itself. Therefore, the SNR constitutes one of the main output pathways of the basal ganglia system regulating mainly voluntary movements (Beckstead et al. 1979).

The mechanisms of GABAergic development in the midbrain have been, surprisingly, neglected until recently. On the one hand, Nakatani et al. (2007) studied the spatial patterning relevant to the GABAergic neurogenesis. Seven distinct progenitor domains were identified along the midbrain neuroepithelium dorsoventral axis (m1–m7; Nakatani et al. 2007; renamed in Puelles et al. 2012). GABAn are originated from the domains m3 to m5 (corresponding to alar ventro-lateral, basal lateral and basal intermediate domains; Puelles et al. 2012), and later in development also from m1 and m2 (corresponding to alar dorsal and alar lateral domains).

On the other hand, Achim et al. (2012) analyzed molecular regulation of ventral tegmental area (VTA) and SNR GABAn differentiation. They demonstrated that GABAn of these regions, mainly the caudal portion, were originated in rhombomere 1 (r1) and occupied their final destination by tangential migration. Nevertheless, the origin of the main rostral mes-diencephalic SNR GABAn population was not described.

Our previous data pointed out that *Nkx6.2* transcription factor plays an important role in the determination and differentiation of the mesencephalon and diencephalon ventral neuronal populations. We found a *Nkx6.2* dynamic expression pattern in the developing mes-diencephalic basal plate, with an early alar positive ventricular domain. However, later in development, only the pre-Edinger-Westphal remains *Nkx6.2* positive (preEW; described previously as Interstitial mesencephalic nucleus by Moreno-Bravo et al. 2010). In other regions of the brain, *Nkx6.2* positive ventricular territories give rise to a massive amount of derivatives which switch off its expression as they differentiate and migrate tangentially (Fogarty et al. 2007). This study prompted us to analyze the fate of the mesencephalic *Nkx6.2* derivatives. We found out that they contribute to several basal populations, being the SNR among them. With the aim to verify their mesencephalic origin, we selected *Six3*, a positive marker of SNR (Conte et al. 2005). This transcription factor belongs to the sine oculis family (Oliver et al. 1995) and it already has been involved in GABAn development (Virolainen et al. 2012). It has a complex expression pattern restricted to the fore- and midbrain (Conte et al. 2005). Summarizing, our working hypothesis postulates a complex multiple origin of the SNR neurons. We demonstrate, using the transcription factors *Nkx6.2* and *Six3*, the mesencephalic neuronal contribution to the SNR. The GABAn generated in the *Nkx6.2* positive ventricular domain populated the SNR in a rostrocaudal gradient.

## Results

### *Nkx6.2* alar derivatives contribute to SNR

**Fig. 1:**
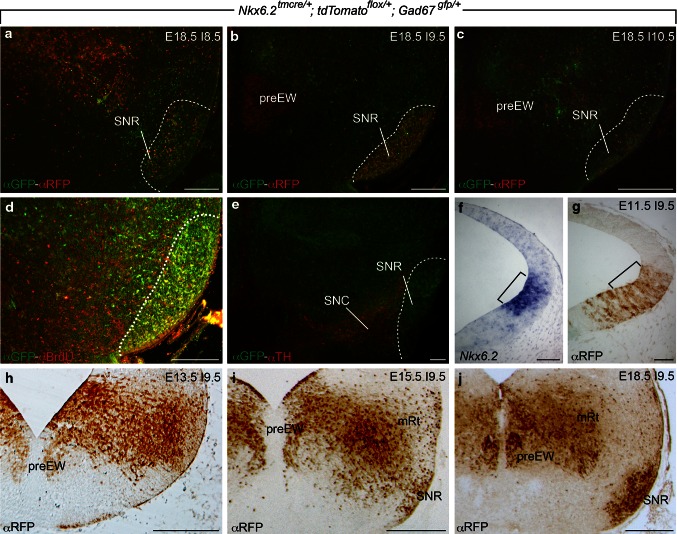
Selected mesencephalic transversal paraffin section through *Nkx6.2* ^*tmcre/*+^; *tdTomato* ^*flox/*+^; *Gad67* ^*gfp/*+^ embryos. **a**–**c** E18.5 sections immunoreacted against α-RFP in *red* (*Nkx6.2* derivatives) and α-GFP in *green* (GABAn). Tamoxifen induced at E8.5, E9.5 and E10.5, respectively. **d** E18.5 section labeled against α-BrdU in *red* and α-GFP in *green*. **e** E18.5 section immunoreacted against α-TH in *red* and α-GAD67 in *green*. **f**, **g** E11.5 induced at E9.5 sections hybridized with *Nkx6.2* probe and immunoreacted against α-RFP, respectively. The *bracket* labels the *Nkx6.2* positive ventricular domain. The *dotted line* indicates the area of SNR. *preEW* pre-Edinger-Westphal, *mRt* mesencephalic reticular formation, *SNC* Substantia nigra pars compacta, *SNR* Substantia nigra pars reticulata. *Scale bars* 250 µm in **a**–**e** and **h**–**j**; 100 µm in **f**, **g**

To study the behavior of *Nkx6.2* GABAergic derivatives in the midbrain and diencephalon, we used the *Nkx6.2* ^*tmcre/*+^; *tdTomato* ^*flox/*+^; *Gad67* ^*gfp/*+^ transgenic mouse. In these mice, all the neurons that were generated from *Nkx6.2* positive progenitors were labeled in red fluorescent color, the GABAn in green fluorescent color and the *Nkx6.2* derived GABAergic neurons in yellow fluorescent color. First, we analyzed along embryonic development the contribution of *Nkx6.2* derivatives to the SNR. Our data allowed us to determine the time window of this process between E8.5 and E10.5. In E18.5 embryos induced by tamoxifen at E8.5, we detected a small number of *Nkx6.2* GABAn in the SNR (Fig. 1a). In embryos induced at E9.5, we found a huge increment in the number of double positive neurons (Fig. 1b). The induction one day later showed only some scattered double positive neurons (Fig. 1c). Therefore, we demonstrated that the peak of *Nkx6.2* contribution to the SNR takes place at E9.5. These data were corroborated by the analysis of long-pulse BrdU labeling at E9.5 (Achim et al. 2012). The proliferating neuroblasts at E9.5 were fated to become SNR GABAergic neurons (Fig. 1d). We used immunohistochemistry for tyrosine hydroxylase to detect the dopaminergic neurons of the SNC and confirm the location of the green fluorescent protein (GFP) positive GABAn in the SNR (Fig. 1e).

The positive *Nkx6.2* neuroblasts switch off its expression as they differentiate and migrate into the mantle layer. The use of the *Nkx6.2* ^*tmcre/*+^ allowed us to label permanently the *Nkx6.2* derivatives (note the difference between *Nkx6.2* expression and *Nkx6.2* derivatives at E11.5, bracket in Fig. 1f, g) and, therefore, to analyze their contribution to the different neuronal populations. We followed the behavior of *Nkx6.2* derivatives, labeled at E9.5, along development. At E13.5, we observed a dense positive group of cells in the mantle layer (Fig. 1h). The positive neurons that contribute to the preEW (Puelles et al. 2012) appeared in the basomedial territory. At E15.5, we found the mesencephalic reticular formation (mRt) highly colonized by *Nkx6.2* derivatives. We identified the SNR by the superficial location of the red fluorescent protein (RFP) positive neurons; the preEW appeared now clearly defined (Fig. 1i). Finally, at E18.5, the three territories were clearly identified. The SNR displayed a dense group of RFP-positive neurons. The *Nkx6.2* derivatives in the mRt showed the typical net-like organization of this complex population. The preEW appeared also densely colonized (Fig. 1j). As previously described, in the basal mes-diencephalic area, only the preEW contains neurons that retain the *Nkx6.2* expression (Moreno-Bravo et al. 2010).

These results demonstrate that SNR is partially originated from a *Nkx6.2* positive ventricular territory. In addition, we have clearly shown that at E9.5 there is a peak of proliferation and determination of SNR neurons originated in this *Nkx6.2* positive territory.

### Rostrocaudal distribution of *Nkx6.2* derivatives

**Fig. 2:**
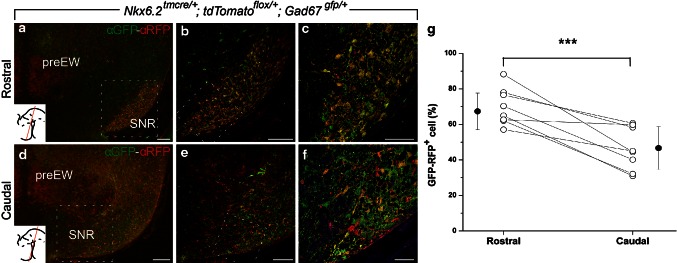
Rostrocaudal distribution of *Nkx6.2* derivatives. Selected mesencephalic transversal paraffin section through a *Nkx6.2* ^*tmcre/*+^; *tdTomato* ^*flox/*+^; *Gad67* ^*gfp/*+^ embryo at E18.5; sections at different levels immunoreacted against α-RFP in *red* (*Nkx6.2* derivatives) and α-GFP in *green* (GABAn). **a** In more rostral sections, there is coexpression between GFP and RFP cells in SNR, but not in other regions, like preEW. *Insets* show the level of the section. **b**, **c** A high magnification to better illustrate the colabelling. **d** In more caudal sections, there is also coexpression between GFP and RFP cells, but in a lower level than in rostral parts. *Insets* show the level of the section. **e**, **f** A high magnification picture of **d**. The *dotted inset* in **a** and **d** indicate the amplified region represented in **b**, **c** and the same in **b**, **c** respect to **e**, **f**. The *graph* represents the significant differences of double-labeled neurons between the two domains, rostral part and caudal part. The values are given as percentage of double labeled (*white points*) and their averages (*black points*). For statistical analysis, Student *t* test was used. *p < 0.0007 for rostral compared with caudal (*n* = 8). For the quantification, a fixed rectangle (275 µm × 687.5 µm) was used. *preEW* pre-Edinger-Westphal, *SNC* Substantia nigra pars compacta, *SNR* Substantia nigra pars reticulata. *Scale bars* 150 µm in **a**, **d**; 100 µm in **b**, **e**; 50 µm in **c**, **f**

The contribution of r1 GABAn to the SNR displays a clear asymmetric distribution along the rostrocaudal axis, being more abundant in the caudal SNR and almost absent in the rostral part (Achim et al. 2012). We analyzed the distribution of the *Nkx6.2* derivatives along this rostrocaudal axis. In mes-diencephalic transversal sections of a *Nkx6.2* ^*tmcre/*+^; *tdTomato* ^*flox/*+^; *Gad67* ^*gfp/*+^ E18.5 embryo induced at E9.5, we detected a gradual distribution of *Nkx6.2* derivatives along the SNR. In rostral sections, we observed a high number of double-labeled neurons tightly packed in the area of the SNR (Fig. 2a–c). In contrast, in caudal sections, we found a low number of double-labeled neurons when compared with the GABAn of the SNR (Fig. 2d–f). Therefore, the GABAergic *Nkx6.2* derivatives are clearly more abundant in the rostral than in the caudal portion of the nucleus (Fig. 2a–f). We quantify this phenomenon selecting a fixed area in rostral and caudal sections of the SNR. The proportion of double-labeled neurons against the total number of GABAn displayed a distribution that clearly proved that the observed phenomenon is statistically significant (Fig. 2g).

Undoubtedly, the SNR is not a homogeneous population. In fact, we have proven that there are clear rostrocaudal differences in the origin of this neuronal nucleus as it was previously suggested (Achim et al. 2012). This diversity in origin could account for functional differences of the SNR neurons (discussed below).

### Tangential migration of SNR subpopulation

**Fig. 3:**
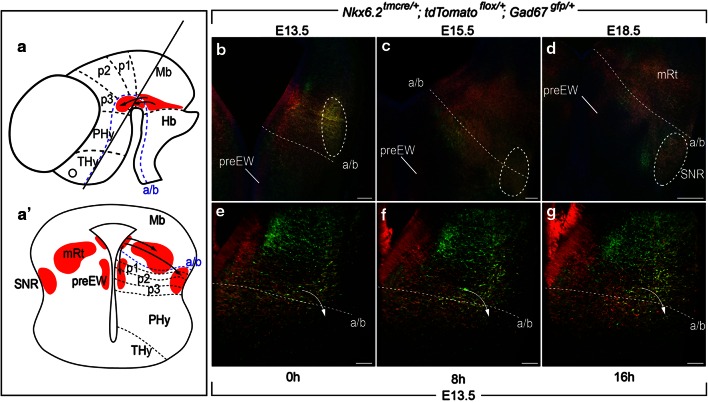
Tangential migration. **a**, **a′** Schematic diagrams of a late stage neural tube (lateral view) and a horizontal slice displaying the tangential migration of *Nkx6.2* derivatives. The *line* indicates the section plane. The *black dotted lines* indicate the boundary of neuromeres. The *blue dotted line* indicates the alar–basal boundary. The *arrows* show the tangential migration of the Nkx6.2 derivatives. Vibratome horizontal sections through *Nkx6.2* ^*tmcre/*+^; *tdTomato* ^*flox/*+^; *Gad67* ^*gfp/*+^ embryos at E13.5 (**b**), E15.5 (**c**) and E18.5 (**d**) immunoreacted against α-RFP in *red* (*Nkx6.2* derivatives) and α-GFP in *green* (GABAn). Static images from a time-lapse experiment where a horizontal section with their endogenous fluorescence was recorded at 0 h (**e**), 8 h (**f**) and 16 h (**g**). The *white dotted lines* indicate the alar–basal boundary. The *arrows* show the tangential migration of SNR *Nkx6.2*-Gabaergic derivatives. *a/b* alar–basal boundary, *Mb* midbrain, *mRt* mesencephalic reticular formation, *Hb* hindbrain, *PHy* peduncular hypothalamus, *preEW* pre-Edinger-Westphal, *p1* pretectum, *p2* thalamus, *p3* prethalamus, *SNR* Substantia nigra pars reticulata, *THy* terminal hypothalamus; *Scale bars* 100 µm in **a**–**c**; 80 µm in **d**–**f**

The alar location of the *Nkx6.2* positive ventricle and the SNR basal situation forced us to study the existence of a tangential migration process. The *Nkx6.2* derivatives must migrate in a rostro-ventral direction to colonize mainly the rostral portion of the SNR (Fig. 3a). Therefore, they not only cross the alar–basal boundary but several interprosomeric limits. We can summarize, in a schematic horizontal section to the diencephalon, the final location of the *Nkx6.2* derivatives. First, close to the ventricle, the tangentially migrated preEW neurons; second, close to the pial surface, the tangentially migrated SNR neurons and finally, in the mantle layer, the radially migrated mRt neurons (Fig. 3a′).

First, we analyzed the behavior of the *Nkx6.2* derivatives through the SNR development using the *Nkx6.2* ^*tmcre/*+^; *tdTomato* ^*flox/*+^; *Gad67* ^*gfp/*+^ strain. At E13.5, the preEW is already well developed. It is located close to the ventricle and distributed along the tegmentum of the diencephalic prosomeres (Fig. 3b). In the mantle layer of the alar midbrain, we localized a compacted group of GABAergic *Nkx6.2* derivatives (Fig. 3b). These double-labeled neurons will later on migrate to the SNR final location. At E15.5, this compacted group is already positioned close to the pial surface and it already started the colonization of the mid-diencephalic basal plate (Fig. 3c). The GABAergic *Nkx6.2* derivatives occupied their final destination at E18.5. They are located close to the pial surface of the basal plate (Fig. 3d). The mRt revealed its complexity with *Nkx6.2* and non-*Nkx6.2* derivatives, GABAergic and non-GABAergic neurons and its distribution along alar and basal domains (Fig. 3d).

Second, we carried out an in vitro time-lapse experiment with an E13.5 horizontal section of a *Nkx6.2* ^*tmcre/*+^; *tdTomato* ^*flox/*+^; *Gad67* ^*gfp/*+^ embryo. Our intention was to follow in real time the tangential migration of the GABAergic *Nkx6.2* derivatives (see video in Online Resource 1). Three static images, at t0, t8 and t16, from the movie clearly illustrate the tangential migration of these precursors from the alar into the basal mantle layer (Fig. 3e–g).

### *Six3* expression in the SNR

**Fig. 4:**
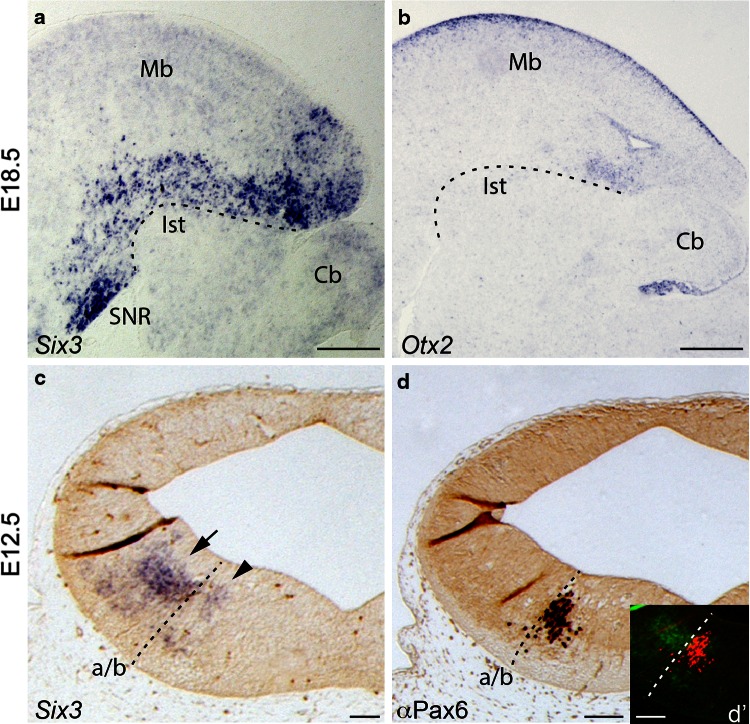
Mesencephalic origin of *Six3* cells. Selected sagittal and transversal paraffin section through *Nkx6.2* ^*tmcre/*+^; *tdTomato* ^*flox/*+^; embryos. **a**, **b** E18.5 sagittal sections hybridized with *Six3* and *Otx2* probes, respectively. *Six3* expression is restricted rostral to the Ist along the midbrain and diencephalon showing a strong expression in the SNR. *Otx2* marks the Ist, delimiting Mb and r1. *Dotted line* in **a** and **b** determine the limit between these regions. **c** E12.5 transversal section with *Six3* in situ hybridization. The *arrow* and *arrowhead* point to the two *Six3* expression domains **d** E12.5 section immunoreacted with antibody against PAX6. **d′** combined **c** and **d**. The Pax6 positive territory separates both domains. *a/b* alar–basal boundary, *Cb* cerebellum, *Ist* isthmus, *Mb* midbrain, *SNR* Substantia nigra pars reticulata; *Scale bars* 300 µm in **a**, **b**; 100 µm in **c**, **d** and **d′**

The analysis of the data obtained suggested a mesencephalic origin of the rostral SNR. The expression of *Nkx6.2* caudal to the isthmic constriction prompted us to search for a transcription factor that could serve as a selective marker of the mesencephalic *Nkx6.2* derivatives (Moreno-Bravo et al. 2012). We decided to use the transcription factor *Six3*, as it already described its expression in the SNR (Conte et al. 2005). It displays a complex expression pattern in the fore- and midbrain and it is never expressed in the hindbrain. In an E18.5, *Six3* expression displayed a sharp boundary between the midbrain and the isthmus (Fig. 4a). This expression coincided with the well-known *Otx2* caudal expression limit (Fig. 4b) that precisely points out the location of the midbrain–hindbrain boundary. In transversal sections to an E12.5 midbrain, *Six3* is expressed in two domains located in the mantle layer. The wider domain is located in the alar plate and the thinner one in the basal plate (arrow and arrowhead, respectively, in Fig. 4c). The *Pax6* positive territory in the basal intermediate region separates both domains (Fig. 4d, d′). The location of the *Six3* positive neurons coincided with the location of the *Nkx6.2* derivatives (compare Fig. 1g with Fig. 4c).

**Fig. 5:**
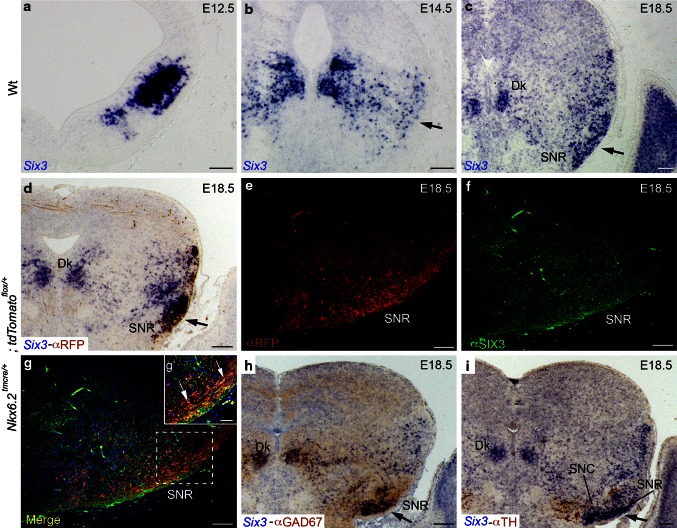
Midbrain *Six3* expression pattern. Selected transversal paraffin sections through embryos at different stages. *Six3* in situ hybridization at E12.5 (**a**), in E14.5 (**b**) and in E18.5 (**c**). The *arrows* in **b** and **c** indicate the area of SNR. **d** E18.5 *Six3* in situ hybridization in blue combined with α-RFP in brown, the *arrow* indicates the SNR. **e**, **f** E18.5 immunofluorescent reacted against α-RFP in *red* (*Nkx6.2* derivatives) and α-SIX3 in *green*. **g** Combined **e** and **f**, showing the coexpression between *Nkx6.2* derivatives and positives *Six3* neurons. **g′** A high magnification of **g** to better illustrate the colabelling, indicated by *arrows*. Cells expressing *Six3* and *Nkx6.2* contribute to the SNR. **h**, **i** α-GAD67 and α-TH in brown, respectively. The *arrows* in **h**, **i** indicate the SNR. *Dk* Darkschewitsch nucleus, *SNC* Substantia nigra pars compacta, *SNR* Substantia nigra pars reticulata. *Scale bars* 100 µm in **a**, **b**, **g′**; 200 µm in **c**–**i**

We analyzed *Six3* mesencephalic expression along embryonic development. At E12.5, we could detect the two domains. Some positive neurons could be found in the superficial mantle layer of the dorsal domain (Fig. 5a). At E14.5, scattered positive neurons were localized in the mantle layer. A group of positive neurons was localized close to the pial surface (arrow in Fig. 5b). At E18.5, the *Six3* positive neurons were localized in their final destination. In the basal medial territory, the Darkschewitsch nucleus was strongly positive for *Six3* (Fig. 5c). A packed group of *Six3* positive neurons was located in the presumptive area of the SNR (arrow in Fig. 5c).

At this last stage, we studied the co-localization of *Six3* with several markers. First, we verified that *Six3* positive cells are present in the SNR region constituted by *Nkx6.2* derivatives (RFP+, arrow in Fig. 5d). We confirmed that SNR *Nx6.2* derivatives also co-expressed *Six3* performing a double immunohistochemistry (Fig. 5e–g,). The SNR RFP-positive neurons co-expressed *Six3* (arrows in Fig. 5g′). Second, we confirmed that these *Six3* positive neurons were GABAergic components of the SNR via GAD67 immunoreactivity (arrow in Fig. 5h). Simultaneously, we corroborated the Dk nucleus identification, as it is a compacted GABAergic basal population (Fig. 5h). Finally, we corroborated through tyrosine hydroxylase immunoreactivity the location of the SNC to prove the correct recognition of the SNR (arrow in Fig. 5i).

Hence, *Six3* is expressed in SNR GABAn *Nkx6.2* derivatives. All these data together, allowed us to confirm that the SNR is partially colonized by GABAn originated in the mesencephalic *Nkx6.2* positive ventricular domain.

## Discussion

The SN GABAn origin is still not completely unveiled despite the studies developed in the last years. Here, we planned to find out the origin of the rostral SNR GABAn. We demonstrated that the rostral SNR is colonized by alar mesencephalic *Nkx6.2* derivatives. These tangentially migrated neurons populate mainly the rostral diencephalic part of the SNR, but are also present in the caudal mesencephalic part.

We hypothesized that the rostral SNR had an alar mesencephalic origin. There were several preliminary data in the literature that supported our hypothesis. Fate map analysis of the mesencephalic basal plate *Shh* positive derivatives demonstrated that the SNR is derived from a *Shh* negative territory (Joksimovic et al. 2009; Achim et al. 2012). Therefore, the generation of the SNR GABAn in the *Shh* negative r1 basal plate or in the mesencephalic alar plate appeared as plausible hypothesis. It has been also proven that the SNR GABAn are partially derived from r1 (Achim et al. 2012). These authors do not exclude a mesencephalic or diencephalic origin of the rostral SNR as the rhombomeric originated GABAn described concentrate in the caudal SNR (Achim et al. 2012).

Our previous studies of *Nkx6.2* expression pattern showed that this transcription factor has a ventricular positive domain adjacent to the alar–basal boundary. This domain gives rise to the preEW, a neuronal population that maintains *Nkx6.2* expression and migrates tangentially into the basal plate. *Nkx6.2* derived neurons usually display tangential migration events. The analysis of the cortical GABA interneurons origin demonstrated that the *Nkx6.2* positive ventricular domain in the medial ganglionic eminence gives rise to a huge amount of GABAn that switch off the expression of the gene as they differentiate and migrate into the cortex (Fogarty et al. 2007). This result supported our hypothesis that the *Nkx6.2* positive ventricular domain could contribute neurons to different mesencephalic populations, by tangential migration, as proposed by Verney et al. 2001.

The use of a transgenic mouse line (*Nkx6.2* ^*tmcre/*+^; *tdTomato* ^*flox/*+^; *Gad67* ^*gfp*+*/*−^) allowed us to label all the derivatives generated from *Nkx6.2* positive neuroblasts (RFP+) and also to distinguish the GABAn (GFP+) among them. This analysis demonstrated the colonization of basal neuronal structures by these derivatives. The SNR was among these neuronal populations. We found RFP+ neurons distributed in a rostrocaudal gradient along the SNR. This distribution was opposed and complementary to the r1 derived GABAn described by Achim et al. (2012).

The distribution of these two subpopulations is translated in neuronal morphological differences, the rostrolateral SNR is populated by fusiform GABAn with major cellular diameter and the caudomedial SNR by elongated GABAn with minor diameter (González-Hernández and Rodríguez 2000). These differences have been also illustrated by SNR projections labeling. Both territories project to the same thalamic areas but the rostral SNR also projects to the centrolateral and thalamic reticular nucleus (Gulcebi et al. 2012).

Due to the proximity of the territories involved (r1, isthmus and midbrain), we confirmed the mesencephalic origin of the RFP+; GFP+ neurons using *Six3* as specific mesencephalic marker. It reported its expression in the SNR (Conte et al. 2005) and it is never expressed along development in the hindbrain (Oliver et al. 1995). Therefore, taking into account of all these data, we can postulate that the SNR GABAn are originated, at least, from two different sources, r1 and midbrain. The GABAn originated in these two territories are distributed in two opposite rostrocaudal gradients and certainly present neuronal morphological, projections and functional differences.

The molecular regulation of the GABAn differentiation associated with the populations described is distinct from the rest of mesencephalic GABAn (Lahti et al. 2013). In the *Gata2* ^*cko*^ mutant, all the midbrain GABAn populations were transformed to a glutamatergic phenotype, except for the SNR and mRf (Lahti et al. 2013). This information together with our data allowed us to postulate that *Nkx6.2* and *Six3* must participate sequentially in the genetic cascade responsible of rostral SNR and mRf neuronal differentiation program.

It has been described that the GABAn development in the different regions of the central nervous system is regulated by diverse genetic mechanisms. Transcription factors, such as *Ascl1*, *Helt* or *Gata2*, have been shown to be selectively required for the development of midbrain GABAn. However, GABAn associated with the dopaminergic nuclei in the VTA and SN do not require any of them (Peltopuro et al. 2010). Indeed, as they develop independently of the known transcriptional regulators, the VTA and SNR GABAn appear molecularly distinct (Guimera et al. 2006; Kala et al. 2009) and therefore likely to have a different origin.

Strikingly, in the *Gata2* ^*cko*^ mutant, all the midbrain GABAn subpopulations were transformed to a glutamatergic phenotype, except for the GABAn associated with the DA neurons in the VTA and SNR, indicating that the remaining mesencephalic GABAn could be born in a region of the midbrain that does not require *Gata2* (Kala et al. 2009). During postmitotic differentiation, *Gata2* controls the expression of downstream GABAn-specific genes and transcription factors (Virolainen et al. 2012), but in *Gata2* ^*cko*^ embryo the expression of *Six3* is altered but does not disappear (Peltopuro et al. 2010).

In the last years, *Tal2* has been identified as a firm candidate to control the differentiation of the SNR GABAn. Together with *Gata2*, it is expressed in all GABAergic precursors in the area spanning from zona limitans to the midbrain–hindbrain boundary (Achim and Salminen 2014). This coexpression does not imply a direct interaction since *Tal2* expression does not require *Gata2* function (Virolainen et al. 2012). The analysis of the *Tal2* lack of function corroborated its role in SNR GABAn differentiation. In the *Tal2* mutant, the *Six3* expression is completely lost and *Gad1* expression, and therefore GABAn differentiation, is absent specifically in the BL domain of the midbrain (location of the *Nkx6.2*+ ventricular domain; Achim et al. 2013). As expected, the generation of the SNR is strongly affected.

All this data support the hypothesis that *Tal2* regulates the differentiation of the SNR GABAn. This regulation takes place in the BL mesencephalic domain where *Nkx6.2* is expressed in the ventricular neuroblasts and *Six3* is expressed in the early-differentiated neurons in the mantle layer. These early GABAn migrate tangentially until their final destination in the SNR.

Another important conclusion to highlight from our data is that we have identified an alar ventricular domain in the mesencephalon able to give rise to different neuronal types. Early in development, it produces glutamatergic neurons that tangentially colonize the preEW nucleus. Later, the *Nkx6.2* positive neuroblasts switch and generate GABAn that tangentially and radially populate the SNR and mRf, respectively.

Finally, the midbrain dopaminergic neurons (SNC and VTA) and their development have been under intensive research due to their relation to Parkinson’s disease. However, importance of the VTA- and SN-associated GABAn for the activity of dopaminergic pathways and behavioral control has become increasingly evident (Vargas-Perez et al. 2009). In fact, GABAn in the ventral mesodiencephalic region are highly important for the function of dopaminergic pathways that regulate multiple aspects of behavior and movement control. These complex morphological and functional structures display intricate developmental processes with multiple origins and migratory routes. Consequently, all our results contribute to implement our knowledge of how these important GABAergic populations are generated.

## Material and methods

### Mouse strains

The mouse lines used and their genotyping have been described previously: *Nkx6.2 cre ER* ^*T2*^ (Feil et al. 1997; Sousa et al. 2009), *GAD67*-*GFP* (Tamamaki et al. 2003), *R26R*-*CAG*-*tdTomato,* obtained from Jackson Laboratories (strain 007905). A loxP-flanked STOP cassette prevents transcription of the downstream RFP variant (tdTomato) in the TdTomato reporter mice.

*Nkx6.2* ^*cre/*+^; *tdTomato* ^*flox/*+^; are generated by crossing homozygous mouse males (Nkx6.2^cre/cre^) with homozygous reporter females (*tdtomato* ^***flox/flox***^). The triple mutant embryos, *Nkx6.2* ^*cre/*+^; *tdTomato* ^*flox/*+^; *Gad67* ^*gfp/*+^ were generated by crossing homozygous mouse males (*Nkx6.2* ^*cre/cre*^) with double heterozygous females (*tdTomato* ^*flox/*+^; *Gad67* ^***gfp/*+**^). For tamoxifen induction, we administer 4 mg of tamoxifen (Sigma, T-5648) (20 mg/ml dissolved in corn oil, Sigma C-8267) per 30 g of pregnant mouse with a gavage needle.

For staging, the day of vaginal plug was counted as embryonic day 0.5 (E0.5). For immunochemistry and in situ hybridization, embryos were fixed in 4 % paraformaldehyde in PBS overnight and completely dehydrated for storage at −20 °C. Samples were paraffin embedded and sectioned at 7 µm or agarose embedded (1 %) and sectioned at 150 µm.

All mouse experiments were performed according to protocols approved by the Universidad Miguel Hernandez OEP committee.

### Immunohistochemistry and in situ hybridization

IHC was performed as described (Moreno-Bravo et al. 2014). The following antibodies were used: Rabbit α-RFP IgG (MBL Cat. No. PM005; 1:100), Mouse α-GAD67 IgG (Millipore Cat. No. MAB5406; 1:300), Rabbit α-TH IgG (Institute Jacques Boy Cat. No. 268020234; 1:1,000), Rabbit α-PAX2 IgG (Zymed 71-6000; 1:5), Sheep α-BrdU IgG (Abcam ab1893; 1:150), Guinea pig a α-SIX3·IgG (Rockland 200-201-A26; 1:200.)

In situ hybridization analyses on paraffin sections were performed as previously described (Moreno-Bravo et al. 2014) using digoxigenin-labeled RNA probes. Mouse cDNA probes used for in situ hybridization analysis were *Six3* (P. Gruss)*, Gad67* (W. Wurst)*, Nkx6.2* and *Otx2* (A. Simeone).

### Birth dating by BrdU labeling

For detection of the peak of neurogenic proliferation, BrdU was administered intraperitoneally to the pregnant females (3 mg/100 g body weight) every 2 h, for a period of 10 h (five injections in total) starting at desired stages.

### Time lapse

For the time-lapse experiments, the embryos were extracted and dissected in cold PBS. Samples were embedded in low melting point agarose (4 %) and sectioned at 250 µm. The sections were collected using Krebs 1X medium (Krebs, glucose, NaHCO_3_, Hepes 1 M 1 %, penicillin/streptomycin 1 %, Gentamicina 0.2 %) at 4 °C. The selected slice was placed in a polycarbonate membrane (MilliCell PICMORG50) with neurobasal medium and incubated during the experiment (37 °C, 5 % CO_2_).

For confocal imaging, a Leica SPE-II DM5550 laser scanning confocal microscope was used. A TCS-SP2-AOBS laser scanning spectral inverted confocal microscope (fitted with temperature and CO_2_ control; Leica Microsystems) was used for live imaging of brain slice culture. Images were collected every 20 min during 16 h. All the focal planes were merged to visualize the maximum projection. Videos were processed with Imaris and ImageJ software.

### Microscopy and quantification

IHC and ISH staining on paraffin and vibratome sections were visualized under fluorescence automated DM6000B microscope and MZ16FA Fluorescence Stereomicroscope (for wide-field microscopy), running Leica Application Suite (LAS) AF6000 Software (version 2.0.2), equipped with a DFC350-FX (monochrome) or DC500 (color) digital cameras. Images were processed and assembled with Adobe Photoshop software.

For quantification, cells were counted only from the rostrocaudal SNR domain. A fix area (275 µm × 687.5 µm) in this region was used to count GABA and *Nkx6.2* positive neurons and then compare rostrocaudal SNR axis. A standard Student’s *t* test was used for comparing the mean values of the data sets.

## Electronic supplementary material

Below is the link to the electronic supplementary material.

Supplementary material 1 **ESM1**. Maximum intensity z projection through E13.5 *Nkx6.2* ^*tmcre/*+^; *tdTomato* ^*flox/*+^; *Gad67* ^*gfp/*+^ horizontal section. Image stacks were taken every 20 min. The video speed is 3 frames/second. The inset shows a magnification of the area of interest. The arrow points a migrating single neuron. Related still images are shown in Fig. 3f-h. (MP4 13,822 kb)
